# Epidemic cycling in a multi-strain SIRS epidemic network model

**DOI:** 10.1186/s12976-016-0040-7

**Published:** 2016-04-18

**Authors:** Xu-Sheng Zhang

**Affiliations:** Department of Statistics, Modelling and Economics, Centre for Infectious Disease Surveillance and Control, Public Health England, 61 Colindale Avenue, London, NW9 5EQ UK; Medical Research Council Centre for Outbreak Analysis and Modelling, Department of Infectious Disease Epidemiology, Imperial College School of Public Health, Norfolk Place, London, W2 1PG UK

**Keywords:** Competition, Cross-immunity, Cyclical dominance of strains, Infectious diseases, Contact network, Oscillatory epidemics

## Abstract

**Background:**

One common observation in infectious diseases caused by multi-strain pathogens is that both the incidence of all infections and the relative fraction of infection with each strain oscillate with time (i.e., so-called Epidemic cycling). Many different mechanisms have been proposed for the pervasive nature of epidemic cycling. Nevertheless, the two facts that people contact each other through a network rather than following a simple mass-action law and most infectious diseases involve multiple strains have not been considered together for their influence on the epidemic cycling.

**Methods:**

To demonstrate how the structural contacts among people influences the dynamical patterns of multi-strain pathogens, we investigate a two strain epidemic model in a network where every individual randomly contacts with a fixed number of other individuals. The standard pair approximation is applied to describe the changing numbers of individuals in different infection states and contact pairs.

**Results:**

We show that spatial correlation due to contact network and interactions between strains through both ecological interference and immune response interact to generate epidemic cycling. Compared to one strain epidemic model, the two strain model presented here can generate epidemic cycling within a much wider parameter range that covers many infectious diseases.

**Conclusion:**

Our results suggest that co-circulation of multiple strains within a contact network provides an explanation for epidemic cycling.

## Background

Recurrent epidemics are a common behaviour of many endemic infectious diseases [1, 2]. Transmission and spread of infectious diseases depend, in part, on the way and frequency of how people contact with each other. The mass-action law which assumes the homogeneous mixing among individuals has been traditionally employed in modelling contact patterns because of simplicity and mathematical tractability. Based on the mass-action law, however, simple transmission dynamics models cannot predict sustained oscillations in incidence [3, 4]. To explain recurrent epidemics, many complicated aspects of both hosts and infectious agents have been included. For example, seasonal forcing due to external driving changes in host behaviour and/or susceptibility, and the intrinsic mechanisms such as interactions between strains of the infectious agents (for a review see [5]). The models that incorporate different elaborate aspects can generate the oscillatory epidemics under certain, usually restricted, parameter ranges.

The actual contact patterns among people surely deviate from the mass-action law [6, 7]. For example, contact patterns between people may display the characteristics of scale-free networks [8] or small-world networks [9]. A recent study shows that it is the contact heterogeneity, rather than transmission efficiency, that limits the emergence and spread of canine influenza virus [10]. This indicates the crucial role of contact structure in infection transmission and spread. Applying the network frameworks into infectious disease modelling has attracted much theoretical attention and shown some novel features (e.g. [11–15]). Letting infection spread on a homogeneous population with a fixed random network structure, Rozhnova and Nunes [16] illustrate sustained cyclical epidemics within a one strain susceptible-infective-recovered-susceptible (SIRS) model. They show that a combination of intrinsic stochasticity due to a finite population size and spatial correlation due to limited contacts may be enough to produce realistic oscillatory patterns observed in recurrent epidemics. As they observed, however, the phase of sustained oscillations for parameter values that correspond to diseases gets thinner with the number of contacts each individual has (which is defined as the degree in network theory) so quickly that the oscillatory phase disappears once the degree exceeds six.

Another striking characteristic of endemic infectious diseases is the fact that they are mostly caused by multi-strain pathogens and the dominant strain alters between epidemics [5]. For childhood diseases, for example, it might have been traditionally thought that only one strain is involved in each disease. With advanced techniques such as polymerase chain reaction and phylogenetic analysis, it has been now recognised that more than one genotypes (or strains in general sense) are co-circulated in, say, measles [17, 18], chicken pox [19–24], rubella [25, 26], pertussis [27–30], mycoplasma pneumoniae [31], and hand-foot-mouth disease [32]. Further, the accumulative evidence that reinfection does occur in, for instance, measles [33–36], chicken pox [37–40], rubella [41–44] and pertussis [45, 46], mycoplasma pneumoniae [47, 48], and hand-foot-mouth disease [49], indicates that immunity against these childhood diseases that were built through nature infection or vaccination wanes. Many other infectious diseases are also caused by multi-strain pathogens such as cholera, dengue, influenza, malaria, Neisseria meningitides and respiratory syncytial virus infection.

Although the structured network plays an important role in infection transmission and polymorphic infectious diseases are quite common, these two characteristics have not yet been collectively investigated on their potential role in generating sustained epidemic cycling. In this study, we consider a two strain SIRS epidemic model (e.g., [50]) and assume that immunity wanes either because of immune loss within the human body or immune escapement due to changes in the circulating strains. Further, following Rozhnova and Nunes [16], two strains are assumed to co-circulate within a random network of a fixed degree. We investigate how cross-immunity between strains and spatial correlation due to contact structure interplay to produce the epidemic cycling, i.e., the concomitant occurrence of sustained oscillations in the total incidence and the alternation of dominant strains. When dealing with polymorphic pathogens, it is worth pointing out the meanings of “strains”. In empirical studies, strains of a pathogen are usually defined serologically or phenotypically. In theoretical modelling, however, strains have been defined immunologically or genetically [51, 52]. In this study we assume this theoretical tradition to allow the model framework to be widely applicable.

## Methods

Within the two strain SIRS model, the population is classified into eight different compartments and modelled as a random network of a fixed degree *κ*. Individuals are denoted by nodes and contacts between individuals by edges. The epidemic dynamics is determined by the following transmission and transition processes. Susceptible nodes (*S*) become infected with strain *i*, *i* = {1,2}, at rate *λ* through an edge with a node of primary infection *I*_*i*_ or a node of secondary infection *J*_*i*_. Primarily infected nodes (*I*_*i*_) recover at rate *γ* to become fully immune (*R*_*i*_) to the infecting strain *i* and partially so to the other strain. The recovered individuals (*R*_*i*_) lose immunity at rate *σ* to become susceptible again, or become secondarily infected at rate (1-*ψ*)*λ* through an edge with a node of infection (*I*_3*-i*_ or *J*_3*-i*_) to become secondarily infected *J*_3*-i*_, *i* = {1,2}. Here *ψ* reflects the reduction in susceptibility due to the previous exposure to other strain (i.e., cross-immunity). Nodes of secondary infection *J*_*i*_, *i* = {1,2} recover at rate *γ* to become fully immune against all strains (i.e., *R*). Nodes of fully immune (*R*) lose immunity at rate *σ* to become susceptible again. These transitions and transmissions are defined according to the pairs or triplets involved in the process [16, 53]. For simplicity we ignore the clustering in the network (c.f., [15, 53]).

Following Eames and Keeling [53], the numbers of people in eight different statuses are represented by [S], [I_1_], [I_2_], [J_1_], [J_2_], [R_1_], [R_2_], and [R]. The additional mortality caused by the virulence of infections is ignored, and birth and death occur at the same rate *μ* to maintain a constant population size, *N* = [S] + [I_1_] + [I_2_] + [J_1_] + [J_2_] + [R_1_] + [R_2_] + [R]. There are (8 × 7)/2 = 28 heterogeneous pairs within the network in which the two nodes of a pair are of different states. The number of homogenous pairs can be found from these of heterogeneous pairs: e.g., [*RR*]= $\kappa \left(N-{\displaystyle \sum_{Y\ne R}\left[Y\right]}\right)-{\displaystyle \sum_{X\ne R}\left[XR\right]}$ and [*SS*]= $\kappa \left[S\right]-{\displaystyle \sum_{X\ne S}\left[SX\right]}$. The state of the system is defined by seven integers of nodes and 28 integers of heterogeneous pairs. To focus on the impact of spatial correlation (i.e., competition among the limited number of partners) and cross-immunity between strains, two strains are assumed to be antigenically indistinguishable.

Transmission of infection among nodes occurs through pair-link and the change of pairs is determined by the triples. The standard pair approximation SIRS model of two strains is described by a set of 28 + 7 = 35 differential equations,

### Equations describing the changing numbers of nodes

$$ \begin{array}{l}\frac{d}{dt}\left[S\right]=\mu \left(N-\left[S\right]\right)-\lambda \left(\left[S{I}_1\right]+\left[S{J}_1\right]+\left[S{I}_2\right]+\left[S{J}_2\right]\right)+\sigma \left(\left[R\right]+\left[{R}_1\right]+\left[{R}_2\right]\right)\hfill \\ {}\frac{d}{dt}\left[{I}_1\right]=-\left(\mu +\gamma \right)\left[{I}_1\right]+\lambda \left(\left[S{I}_1\right]+\left[S{J}_1\right]\right)\hfill \\ {}\frac{d}{dt}\left[{I}_2\right]=-\left(\mu +\gamma \right)\left[{I}_2\right]+\lambda \left(\left[S{I}_2\right]+\left[S{J}_2\right]\right)\hfill \\ {}\frac{d}{dt}\left[{R}_1\right]=\gamma \left[{I}_1\right]-\left(\mu +\sigma \right)\left[{R}_1\right]-\lambda \left(1-\psi \right)\left(\left[{R}_1{I}_2\right]+\left[{R}_1{J}_2\right]\right)\hfill \\ {}\frac{d}{dt}\left[{R}_2\right]=\gamma \left[{I}_2\right]-\left(\mu +\sigma \right)\left[{R}_2\right]-\lambda \left(1-\psi \right)\left(\left[{R}_2{I}_1\right]+\left[{R}_2{J}_1\right]\right)\hfill \\ {}\frac{d}{dt}\left[{J}_1\right]=\lambda \left(1-\psi \right)\left(\left[{R}_2{I}_1\right]+\left[{R}_2{J}_1\right]\right)-\left(\mu +\gamma \right)\left[{J}_1\right]\hfill \\ {}\frac{d}{dt}\left[{J}_2\right]=\lambda \left(1-\psi \right)\left(\left[{R}_1{I}_2\right]+\left[{R}_1{J}_2\right]\right)-\left(\mu +\gamma \right)\left[{J}_2\right]\hfill \end{array} $$

### Equations describing the changing numbers of pairs

1 $$ \begin{array}{l}\begin{array}{l}\frac{d}{dt}\left[S{I}_1\right]=\lambda \left(\left[SS{I}_1\right]+\left[SS{J}_1\right]-\left[{I}_1S{I}_1\right]-\left[{J}_1S{I}_1\right]-\left[{I}_2S{I}_1\right]-\left[{J}_2S{I}_1\right]\right)-\left(\lambda +\gamma \right)\left[S{I}_1\right]\\ {}+\sigma \left(\left[R{I}_1\right]+\left[{R}_1{I}_1\right]+\left[{R}_2{I}_1\right]\right)+\mu \left(\kappa \left[{I}_1\right]-2\left[S{I}_1\right]\right)\end{array}\hfill \\ {}\begin{array}{l}\frac{d}{dt}\left[S{I}_2\right]=\lambda \left(\left[SS{I}_2\right]+\left[SS{J}_2\right]-\left[{I}_1S{I}_2\right]-\left[{J}_1S{I}_2\right]-\left[{I}_2S{I}_2\right]-\left[{J}_2S{I}_2\right]\right)-\left(\lambda +\gamma \right)\left[S{I}_2\right]\\ {}+\sigma \left(\left[R{I}_2\right]+\left[{R}_1{I}_2\right]+\left[{R}_2{I}_2\right]\right)+\mu \left(\kappa \left[{I}_2\right]-2\left[S{I}_2\right]\right)\end{array}\hfill \\ {}\begin{array}{l}\frac{d}{dt}\left[S{R}_1\right]=-\lambda \left(\left[{I}_1S{R}_1\right]+\left[{J}_1S{R}_1\right]+\left[{I}_2S{R}_1\right]+\left[{J}_2S{R}_1\right]\right)-\lambda \left(1-\psi \right)\left(\left[S{R}_1{I}_2\right]+\left[S{R}_1{J}_2\right]\right)+\gamma \left[S{I}_1\right]\\ {}+\sigma \left(\left[{R}_1{R}_1\right]+\left[{R}_1{R}_2\right]+\left[{R}_1R\right]-\left[S{R}_1\right]\right)+\mu \left(\kappa \left[{R}_1\right]-2\left[S{R}_1\right]\right)\end{array}\hfill \\ {}\begin{array}{l}\frac{d}{dt}\left[S{R}_2\right]=-\lambda \Big(\left[{I}_1S{R}_2\right]+\left[{J}_1S{R}_2\right]+\left[{I}_2S{R}_2\right]+\left[{J}_2S{R}_2\right]-\lambda \left(1-\psi \right)\left(\left[S{R}_2{I}_1\right]+\left[S{R}_2{J}_1\right]\right)+\gamma \left[S{I}_2\right]\\ {}+\sigma \left(\left[{R}_1{R}_2\right]+\left[{R}_2{R}_2\right]+\left[{R}_2R\right]-\left[S{R}_2\right]\right)+\mu \left(\kappa \left[{R}_2\right]-2\left[S{R}_2\right]\right)\end{array}\hfill \\ {}\begin{array}{l}\frac{d}{dt}\left[SR\right]=-\lambda \left(\left[{I}_1SR\right]+\left[{J}_1SR\right]+\left[{I}_2SR\right]+\left[{J}_2SR\right]\right)+\gamma \left(\left[S{J}_1\right]+\left[S{J}_2\right]\right)\\ {}+\sigma \left(\left[{R}_1R\right]+\left[{R}_2R\right]+\left[RR\right]-\left[SR\right]\right)+\mu \left(\kappa \left[R\right]-2\left[SR\right]\right)\end{array}\hfill \\ {}\begin{array}{l}\frac{d}{dt}\left[S{J}_1\right]=\lambda \left(1-\psi \right)\left(\left[S{R}_2{I}_1\right]+\left[S{R}_2{J}_1\right]\right)-\lambda \left(\left[{I}_1S{J}_1\right]+\left[{J}_1S{J}_1\right]+\left[{I}_2S{J}_1\right]+\left[{J}_2S{J}_1\right]\right)-\left(\lambda +\gamma \right)\left[S{J}_1\right]\\ {}+\sigma \left(\left[R{J}_1\right]+\left[{R}_1{J}_1\right]+\left[{R}_2{J}_1\right]\right)+\mu \left(\kappa \left[{J}_1\right]-2\left[S{J}_1\right]\right)\end{array}\hfill \\ {}\begin{array}{l}\frac{d}{dt}\left[S{J}_2\right]=\lambda \left(1-\psi \right)\left(\left[S{R}_1{I}_2\right]+\left[S{R}_1{J}_2\right]\right)-\lambda \left(\left[{I}_1S{J}_2\right]+\left[{J}_1S{J}_2\right]+\left[{I}_2S{J}_2\right]+\left[{J}_2S{J}_2\right]\right)-\left(\lambda +\gamma \right)\left[S{J}_2\right]\\ {}+\sigma \left(\left[R{J}_2\right]+\left[{R}_1{J}_2\right]+\left[{R}_2{J}_2\right]\right)+\mu \left(\kappa \left[{J}_2\right]-2\left[S{J}_2\right]\right)\end{array}\hfill \\ {}\frac{d}{dt}\left[{I}_1{I}_2\right]=\lambda \left(2\left[{I}_1S{I}_2\right]+\left[{J}_1S{I}_2\right]+\left[{I}_1S{J}_2\right]\right)-2\left(\gamma +\mu \right)\left[{I}_1{I}_2\right]\hfill \\ {}\frac{d}{dt}\left[{I}_1{R}_1\right]=\lambda \left(\left[{I}_1S{R}_1\right]+\left[{J}_1S{R}_1\right]\right)-\lambda \left(1-\psi \right)\left(\left[{I}_1{R}_1{I}_2\right]+\left[{I}_1{R}_1{J}_2\right]\right)+\gamma \left[{I}_1{I}_1\right]-\left(\sigma +\gamma +2\mu \right)\left[{I}_1{R}_1\right]\hfill \\ {}\frac{d}{dt}\left[{I}_1{R}_2\right]=\lambda \left(\left[{I}_1S{R}_2\right]+\left[{J}_1S{R}_2\right]\right)-\lambda \left(1-\psi \right)\left(\left[{I}_1{R}_2{I}_1\right]+\left[{I}_1{R}_2{J}_1\right]+\left[{I}_1{R}_2\right]\right)+\gamma \left[{I}_2{I}_1\right]-\left(\sigma +\gamma +2\mu \right)\left[{I}_1{R}_2\right]\hfill \\ {}\frac{d}{dt}\left[{I}_1R\right]=\lambda \left(\left[{I}_1SR\right]+\left[{J}_1SR\right]\right)+\gamma \left(\left[{I}_1{J}_1\right]+\left[{I}_1{J}_2\right]\right)-\left(\sigma +\gamma +2\mu \right)\left[{I}_1R\right]\hfill \\ {}\frac{d}{dt}\left[{I}_1{J}_1\right]=\lambda \left(\left[{I}_1S{J}_1\right]+\left[{J}_1S{J}_1\right]+\left[S{J}_1\right]\right)+\lambda \left(1-\psi \right)\left(\left[{I}_1{R}_2{I}_1\right]+\left[{I}_1{R}_2{J}_1\right]+\left[{I}_1{R}_2\right]\right)-2\left(\gamma +\mu \right)\left[{I}_1{J}_1\right]\hfill \\ {}\frac{d}{dt}\left[{I}_1{J}_2\right]=\lambda \left(\left[{I}_1S{J}_2\right]+\left[{J}_1S{J}_2\right]\right)+\lambda \left(1-\psi \right)\left(\left[{I}_1{R}_1{I}_2\right]+\left[{I}_1{R}_1{J}_2\right]\right)-2\left(\gamma +\mu \right)\left[{I}_1{J}_2\right]\hfill \\ {}\frac{d}{dt}\left[{I}_2{R}_1\right]=\lambda \left(\left[{I}_2S{R}_1\right]+\left[{J}_2S{R}_1\right]\right)-\lambda \left(1-\psi \right)\left(\left[{I}_2{R}_1{I}_2\right]+\left[{I}_2{R}_1{J}_2\right]+\left[{I}_2{R}_1\right]\right)+\gamma \left[{I}_2{I}_1\right]-\left(\sigma +\gamma +2\mu \right)\left[{I}_2{R}_1\right]\hfill \\ {}\frac{d}{dt}\left[{I}_2{R}_2\right]=\lambda \left(\left[{I}_2S{R}_2\right]+\left[{J}_2S{R}_2\right]\right)-\lambda \left(1-\psi \right)\left(\left[{I}_2{R}_2{I}_1\right]+\left[{I}_2{R}_2{J}_1\right]\right)+\gamma \left[{I}_2{I}_2\right]-\left(\sigma +\gamma +2\mu \right)\left[{I}_2{R}_2\right]\hfill \\ {}\frac{d}{dt}\left[{I}_2R\right]=\lambda \left(\left[{I}_2SR\right]+\left[{J}_2SR\right]\right)+\gamma \left(\left[{J}_1{I}_2\right]+\left[{J}_2{I}_2\right]\right)-\left(\sigma +\gamma +2\mu \right)\left[{I}_2R\right]\hfill \\ {}\frac{d}{dt}\left[{I}_2{J}_1\right]=\lambda \left(\left[{I}_2S{J}_1\right]+\left[{J}_2S{J}_1\right]\right)+\lambda \left(1-\psi \right)\left(\left[{I}_2{R}_2{I}_1\right]+\left[{I}_2{R}_2{J}_1\right]\right)-2\left(\gamma +\mu \right)\left[{I}_2{J}_1\right]\hfill \\ {}\frac{d}{dt}\left[{I}_2{J}_2\right]=\lambda \left(\left[{I}_2S{J}_2\right]+\left[{J}_2S{J}_2\right]+\left[S{J}_2\right]\right)+\lambda \left(1-\psi \right)\left(\left[{I}_2{R}_1{I}_2\right]+\left[{I}_2{R}_1{J}_2\right]+\left[{I}_2{R}_1\right]\right)-2\left(\gamma +\mu \right)\left[{I}_2{J}_2\right]\hfill \\ {}\frac{d}{dt}\left[{R}_1{R}_2\right]=-\lambda \left(1-\psi \right)\left(\left[{R}_1{R}_2{I}_1\right]+\left[{R}_1{R}_2{J}_1\right]+\left[{I}_2{R}_1{R}_2\right]+\left[{J}_2{R}_1{R}_2\right]\right)+\gamma \left(\left[{I}_1{R}_2\right]+\left[{R}_1{I}_2\right]\right)-2\left(\sigma +\mu \right)\left[{R}_1{R}_2\right]\hfill \\ {}\frac{d}{dt}\left[{R}_1R\right]=-\lambda \left(1-\psi \right)\left(\left[{I}_2{R}_1R\right]+\left[{J}_2{R}_1R\right]\right)+\gamma \left(\left[{I}_1R\right]+\left[{R}_1{J}_1\right]+\left[{R}_1{J}_2\right]\right)-2\left(\sigma +\mu \right)\left[{R}_1R\right]\hfill \\ {}\frac{d}{dt}\left[{R}_1{J}_1\right]=\lambda \left(1-\psi \right)\left(\left[{R}_1{R}_2{I}_1\right]+\left[{R}_1{R}_2{J}_1\right]-\left[{I}_2{R}_1{J}_1\right]-\left[{J}_2{R}_1{J}_1\right]\right)+\gamma \left[{I}_1{J}_1\right]-\left(\gamma +\sigma +2\mu \right)\left[{R}_1{J}_1\right]\hfill \\ {}\frac{d}{dt}\left[{R}_1{J}_2\right]=\lambda \left(1-\psi \right)\left(\left[{R}_1{R}_1{I}_2\right]+\left[{R}_1{R}_1{J}_2\right]-\left[{I}_2{R}_1{J}_2\right]-\left[{J}_2{R}_1{J}_2\right]-\left[{R}_1{J}_2\right]\right)+\gamma \left[{I}_1{J}_2\right]-\left(\gamma +\sigma +2\mu \right)\left[{R}_1{J}_2\right]\hfill \\ {}\frac{d}{dt}\left[{R}_2R\right]=-\lambda \left(1-\psi \right)\left(\left[{I}_1{R}_2R\right]+\left[{J}_1{R}_2R\right]\right)+\gamma \left(\left[{I}_2R\right]+\left[{R}_2{J}_2\right]+\left[{R}_2{J}_1\right]\right)-2\left(\sigma +\mu \right)\left[{R}_2R\right]\hfill \\ {}\frac{d}{dt}\left[{R}_2{J}_1\right]=\lambda \left(1-\psi \right)\left(\left[{R}_2{R}_2{I}_1\right]+\left[{R}_2{R}_2{J}_1\right]-\left[{I}_1{R}_2{J}_1\right]-\left[{J}_1{R}_2{J}_1\right]-\left[{R}_2{J}_1\right]\right)+\gamma \left[{I}_2{J}_1\right]-\left(\gamma +\sigma +2\mu \right)\left[{R}_2{J}_1\right]\hfill \\ {}\frac{d}{dt}\left[{R}_2{J}_2\right]=\lambda \left(1-\psi \right)\left(\left[{R}_2{R}_1{I}_2\right]+\left[{R}_2{R}_1{J}_2\right]-\left[{I}_1{R}_2{J}_2\right]-\left[{J}_1{R}_2{J}_2\right]\right)+\gamma \left[{I}_2{J}_2\right]-\left(\gamma +\sigma +2\mu \right)\left[{R}_2{J}_2\right]\hfill \\ {}\frac{d}{dt}\left[R{J}_1\right]=\lambda \left(1-\psi \right)\left(\left[R{R}_2{I}_1\right]+\left[R{R}_2{J}_1\right]\right)+\gamma \left(\left[{J}_1{J}_2\right]+\left[{J}_1{J}_1\right]\right)-\left(\gamma +\sigma +2\mu \right)\left[R{J}_1\right]\hfill \\ {}\frac{d}{dt}\left[R{J}_2\right]=\lambda \left(1-\psi \right)\left(\left[R{R}_1{I}_2\right]+\left[R{R}_1{J}_2\right]\right)+\gamma \left(\left[{J}_1{J}_2\right]+\left[{J}_2{J}_2\right]\right)-\left(\gamma +\sigma +2\mu \right)\left[R{J}_2\right]\hfill \\ {}\frac{d}{dt}\left[{J}_1{J}_2\right]=\lambda \left(1-\psi \right)\left(\left[{I}_1{R}_2{J}_2\right]+\left[{J}_1{R}_2{J}_2\right]+\left[{J}_1{R}_1{I}_2\right]+\left[{J}_1{R}_1{J}_2\right]\right)-2\left(\gamma +\mu \right)\left[{J}_1{J}_2\right]\hfill \end{array} $$

Here [*XYZ*] represents the number of triple *XYZ* with node *Y* having contacts with both *X* and *Z*. To close the system, the number of triples is approximated in terms of the number of pairs as in [53],

2 $$ \left[XYZ\right]\approx \frac{k-1}{k}\frac{\left[XY\right]\left[YZ\right]}{\left[Y\right]} $$

Noting that there are 15 × 8 = 120 transmission and transition processes in the two strain SIRS model, the above eqs. (1, 2) can also be obtained by a coarse description where the effect of the change in state of a given node on the *κ* pairs that it forms is averaged over each pair type [16].

The complexity of the two strain dynamics allows us to investigate the combined effects of cross-immunity and competition between two strains on dynamic patterns of endemic infectious diseases, along with spatial correlation embedded within the random network. To ignore the stochasticity due to the limited size of population, here we consider a human population of a very large size *N*. The fourth order Runge-Kutta algorithm is used to solve the eqs. (1, 2) and the programme is coded in R3.2.0 [54] to simulate the dynamical process.

## Results and discussion

**Fig. 1:**
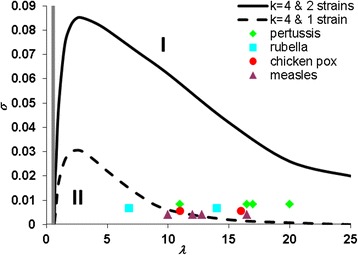
Phase diagram in the (λ,σ) plane for pair approximation model of two strain SIRS model. Other parameters: *κ* = 4, *μ* = 0.0005, and *ψ* = 0.0. The boundary of the one strain model of [16] is included for comparison. Region I is the constant endemics phase where the number of new infections is balanced by the number of recoveries and region II the oscillatory epidemics phase. The critical infection rate that separates disease-free phase and region I is λ_c_ ≈ 0.5. Data for the four childhood infectious diseases are from [16]. As the infectious period is used as the unit of time, the birth and death rate of *μ* = 0.0005 is equivalent to a life span of about 50 years in the model system for infectious diseases which have infectious periods of 1-2 weeks (such as Measles, chickenpox, rubella). It is worth mentioning that our predicted threshold waning rate of immunity in one strain SIRS model with *μ* = 0.0005 (i.e., the dashed line) are only slightly smaller than that presented in [16] who do not consider the birth rate

For simplicity, the time scale is set so that *γ* =1 (i.e. the average infectious duration is taken as the time unit). The numerical calculations show that the final dynamic patterns of epidemic time series are independent of the initial conditions. The phase diagram of the two strain SIRS model is shown in Fig. 1, which is divided into three parts as that for one strain model (see Fig. 1 of [16]). When infection rate λ is less than a critical value *λ*_*c*_ ≈ (*σ* + 1)/[(*κ* − 2) + *σ*(*κ* − 1)] from [16], disease cannot survive (disease-free phase). For a given infection rate λ that is larger than λ_c_, only a steady endemic with constant incidence is possible if immunity waning rate *σ* is larger than a critical value *σ*_c_ (region I: constant incidence phase); otherwise, the sustained oscillatory epidemic emerges (region II: oscillatory incidence phase). Comparison of our model with another two strain model that assumes homogeneous mixing [50] suggests that the spatial correlation due to network structure induces the sustained epidemic cycling as in the one strain model [16]. From Fig. 1, it is obvious that the critical value *σ*_c_ for the two strain model is much higher than that for the one strain model. (Note that resonant amplification of stochastic fluctuations due to a finite population size can only slightly increase the values of *σ*_c_ in the one strain deterministic model [16]). This indicates that under the circumstance of the same epidemic characteristics, the two strain model allows for oscillatory epidemics in infectious diseases that have much shorter immunity periods, and thus expands model parameter range for oscillatory epidemics.

The published data for childhood infectious diseases that occur recurrently fall into the oscillatory phase of the two strain model (see Fig. 1); comparably, only some infection data are within the oscillatory phase of the one strain model [16]. This difference results from the competition between strains. The competition comes from two different aspects. One is ecological interference [55] that infectiousness with one strain avoids further being infected by another strain as in multi-strain models (e.g. [56]). This acts equivalently as a kind of convalesce with respect to another strain and enhances the emergence of sustained oscillations in incidence [57, 58]. The other is spatial correlation due to contact network structure. The limited number of nodes each node links in the contact network leads to the competition, which increases as the degree *κ* decreases and then induces cyclical epidemics [16]. These two aspects work together to expand greatly the oscillatory phase in the two strain model.

**Fig. 2:**
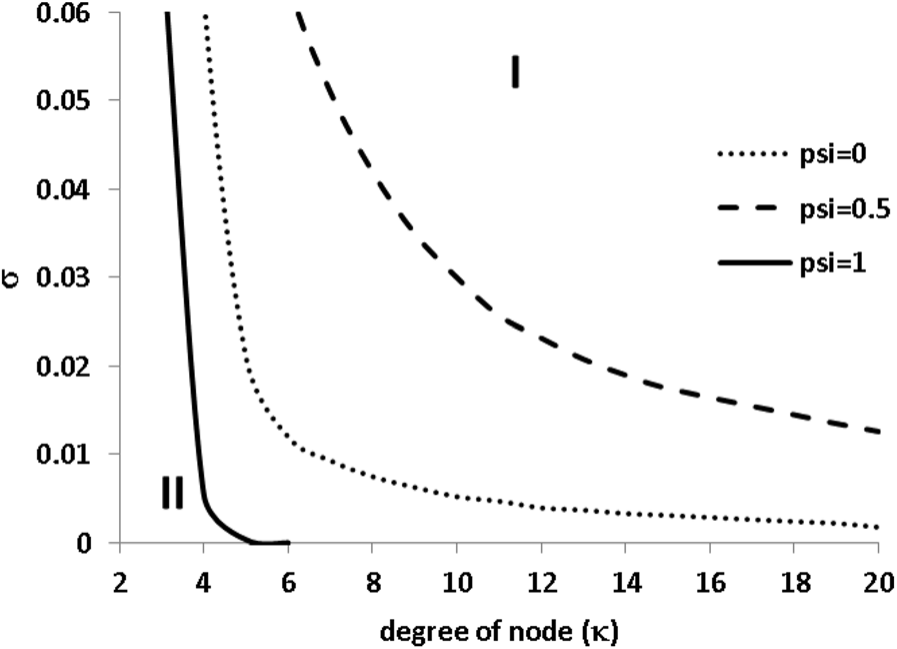
The effect of the degree on the oscillatory phase under three levels of cross-immunity. Region I is for constant endemics where the number of new infections is balanced by the number of recoveries and region II for the oscillatory epidemics. The transmission rate is *λ* = 10 and the birth rate is *μ* = 0.0005

**Fig. 3:**
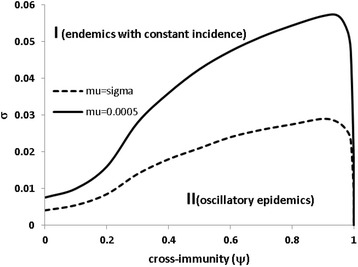
The effect of cross-immunity on the oscillatory phase. The parameter area for cycling epidemics (II) increases with cross-immunity but reduces rapidly when cross-immunity becomes complete. Other parameters: *κ* = 8, λ = 10 and two birth rates are assumed: *μ* = 0.0005 (solid line) and *μ* = σ (dashed line). In the later situation the threshold waning rate of immunity nearly halves, which indicates that most of individuals stay in the recovery and immune compartments

Introduction of cross-immunity between strains further enlarges the oscillatory phase in the two strain model (Figs. 2 and 3). In contrast to the one strain model where oscillatory phase disappears on networks of degree *κ* > 6 [16], the oscillatory epidemics in the two strain model persist on contact networks of a very high degree *κ* (Fig. 2). This implies that the ecological interference and cross-immunity in some ways compensate weakened spatial correlation at highly contact networks. Therefore, the two strain SIRS epidemic model can easily explain the oscillatory behaviours observed in childhood infectious diseases. Under the extreme circumstance of complete cross-immunity, the oscillatory phase decreases considerably (Fig. 3); for the situation shown in Fig. 2 recurrent epidemics emerge only on contact networks of a degree *κ* < 5.

Gupta et al. [59] investigate multi-strain SIR models within a randomly mixing population in which strains can exchange through mutation and recombination. They illustrate that host immunity dictates the structure and dynamics of the pathogen population, with cyclical dominance of non-overlapping sets of strains occurring only at intermediate levels of cross-immunity. Though Buckee et al. [12] show a significant role of host contact network structure in mediating pathogen strain structure and dynamics, qualitatively similar patterns dictated by cross-immunity remains: cyclical epidemics emerges only at the intermediate levels of cross-immunity. Spreading through a random network structure with a fixed degree, however, our two strain SIRS epidemics show cyclical epidemics even under circumstances of no cross-immunity (Figs. 1, 2 and 3) and of complete cross-immunity (Figs. 2 and 3). The difference comes from that, comparing to the SIR models of [12, 59], here we consider waning immunity which takes place through both immunity loss and immunity escapement. Although we do not explicitly model the continuous changes in two strains, these changes have been taken into and reflected in the waning immunity of our model. The models of Gupta et al. [59] and Buckee et al. [12] do not assume immunity loss in human body; however, immunity escapement occurs due to the continuous exchanges in strains through mutation and recombination. Under the two extreme circumstances: complete cross-immunity wherein there is no immunity escapement, and no cross-immunity wherein all strains become independently and act as a single strain, it is a quite straightforward result that no recurrent epidemics will be generated within their models. This is compatible with the pattern shown in Fig. 2: When each individual can contact many others, our model will also prohibit recurrent epidemics under the two extreme circumstances.

**Fig. 4:**
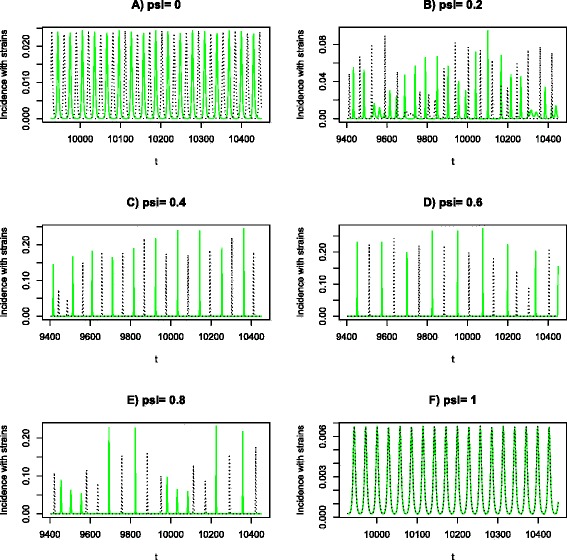
Examples of incidence time series under eight different levels of cross-immunity. Other parameters: *κ* = 4, *λ* = 10, *σ* = 0.005, and *μ* = 0.0005. Two strains are represented by different lines. In graph (**f**) *ψ* = 1, two strains completely synchronize so that their incidences overlap

Without cross-immunity, our model demonstrates that the total incidence oscillates and two strains anti-synchronize as shown in Fig. 4a, which can be understood as a consequence of competition between strains and spatial correlation of nodes within the random network as argued above (cf., [58, 59]). On another extreme situation of full cross-immunity (*ψ* = 1.0), the total incidence oscillates but two strains synchronizes as shown in Fig. 4f. This is in agreement with the conclusions from [5] who consider complete cross-immune strains within a well-mixing population. However, the underlying mechanisms for oscillatory epidemics are different. In this study it is due to the interplay of competition and spatial correlation in contact structure while in [5], it is due to the enhanced infectivity within concurrent infection. With intermediate levels of cross-immunity, recurrent epidemics oscillate irregularly and dominant strains alternate between epidemics (Figs. 4b–e; cf., [12, 59]).

Three possible interactions have been examined for sustained oscillatory epidemics: ecological interference which arises from the exclusion of strain during the infectious period [56–58], and immune-mediated competition which takes into effect during the immunity period [12, 59], network-mediated spatial correlation which makes the whole population in some way act as many spatial subpopulations [16]. It is worth pointing out the difference between the first two interactions. The ecological interference occurs during the infectious period and because of the removal of individuals from the susceptible pool during the period of being infected with one strain (see [55, 60]). The immune-mediated competition (i.e., cross-immunity) takes place when individuals recover from infection with one strain and then become immune against the infecting strain and also (partially) immune against another strain during the immunity period. As demonstrated, each of these together with other factors can induce oscillatory epidemics. However, the conditions for oscillatory epidemics appear to be quite restricted. The three interactions act collectively in our two strain SIRS epidemic model and epidemic cycling is shown to emerge in a much less restrictive parameter space.

Although we focus on a system of two strains, the results of this study are generally applicable to the multi-strain setting. Technically, it is not trivial to write down the differential equations even for the epidemic system caused by three strains; however, the logical reason for this is simply that with more strains co-circulating within the same network, the three interactions just discussed will increase, which consequently facilitates the emergence of recurrent epidemics. In this study, we show the potential of SIRS epidemic models that allow multiple strains to co-circulate within a network structural population to generate recurrent epidemics. Although childhood diseases were used in Fig. 1 as examples to demonstrate how our two strain model makes recurrent epidemics more likely than the one strain model of Rozhnova and Nunes [16], the model framework presented here should be also applicable to other infectious diseases caused by multi-strain pathogens. To explore the specific mechanism for a particular infectious disease caused by multiple strains, however, we need to estimate the exact values of model parameters by fitting the model outputs to the empirical dynamical patterns observed in human populations such as the duration and size of epidemics and the gap between epidemics (as in [61]). This is the issue we will aim at in future.

## Conclusion

We consider the pair approximation of a two strain SIRS epidemic model in a host population with every individual in contact with a fixed number of other individuals. We show that interactions between strains due to ecological interference and limited contacts within a network structured population can induce sustained oscillatory patterns in both total incidence and dominant strains. Though our model is simplified in that clustering and heterogeneity in contact network and stochasticity have been neglected, inclusion of these more realistic aspects will facilitate the diversity [12, 15] and fluctuation [16, 62] and therefore cannot change our conclusion. Our results suggest another possible mechanism for the observed epidemic cycling: interplay of spatial correlation due to contact network and interactions between strains through exclusion of other infection during the infectious period and immune protection during the immunity period (cf., [5]).
